# Genotypic and Phenotypic Characterisation of Enteroaggregative *Escherichia coli* from Children in Rio de Janeiro, Brazil

**DOI:** 10.1371/journal.pone.0069971

**Published:** 2013-07-30

**Authors:** Fernanda L. S. França, Timothy J. Wells, Douglas F. Browning, Raquel Tayar Nogueira, Felipe Silva Sarges, Ana Claudia Pereira, Adam F. Cunningham, Kely Lucheze, Ana Claudia Paula Rosa, Ian R. Henderson, Maria das Graças de Luna

**Affiliations:** 1 Departamento de Microbiologia, Imunologia e Parasitologia, Faculdade de Ciências Médicas, Universidade do Estado do Rio de Janeiro, Rio de Janeiro, Brazil; 2 Institute of Microbiology and Infection, University of Birmingham, Edgbaston, Birmingham, United Kingdom; Centre National de la Recherche Scientifique, Aix-Marseille Université, France

## Abstract

Enteroaggregative *Escherichia coli* (EAEC) is a significant cause of diarrhoeal illness in both children and adults. Genetic heterogeneity and recovery of EAEC strains from both healthy and diseased individuals complicates our understanding of EAEC pathogenesis. We wished to establish if genetic or phenotypic attributes could be used to distinguish between strains asymptomatically colonising healthy individuals and those which cause disease. Genotypic screening of a collection of twenty four EAEC isolates from children with and without diarrhoea revealed no significant differences in the repertoire of putative virulence factors present in either group of strains. In contrast, EAEC strains from phylogroup A were more strongly associated with asymptomatic groups whereas strains from phylogroup D were more associated with cases of diarrhoea. Phenotypic screening revealed no differences in the ability of strains from either cohort of children to form biofilms, to adhere to and invade cells in tissue culture or to cause disease in the *Caenorhabditis elegans* model of infection. However, the latter assay did reveal significant reduction in nematode killing rates when specific virulence factors were deleted from human pathogenic strains. Our results suggest that current models of infection are not useful for distinguishing avirulent from pathogenic strains of EAEC but can be useful in studying the effect of specific virulence factors.

## Introduction

Based on genotypic and phenotypic traits diarrhoeagenic *Escherichia coli* can be categorised into six different pathotypes: enteroaggregative *E. coli* (EAEC), enterotoxigenic *E. coli* (ETEC), enteroinvasive *E. coli* (EIEC), enteropathogenic *E. coli* (EPEC), enterohaemorrhagic *E. coli* (EHEC) and diffuse-adhering *E. coli* (DAEC). EAEC, which is defined by a characteristic “stacked-brick” aggregative adherence (AA) to cultured HEp-2 cells [1], was first recognised as a separate group from other diarrhoeagenic *E. coli* in 1987 [2], [3]. Initially associated with persistent infantile diarrhoea, EAEC is now recognised as a cause of acute and persistent diarrhoea in HIV-positive adults, in travellers and in large outbreaks in the industrialised world [2], [3]. Several studies have reported that EAEC are responsible for the majority of *E. coli*-mediated diarrhoea in the developed world [3] and the importance of EAEC as a pathogen was highlighted by the shiga-toxin producing EAEC O104:H4 outbreak in Germany which affected more than 4000 individuals [4].

Despite the importance of EAEC as a pathogen this pathotype is not preferentially included during clinical diagnostic tests [5] and the molecular mechanisms underlying pathogenesis are poorly understood. Early molecular studies of prototypical strains revealed that the AA phenotype was due to the presence of plasmids, collectively called pAA [6]. The pAA plasmids carry many factors associated to EAEC pathogenesis, including the heat-stable toxin-1 (EAST-1) [7], fimbrial adhesins (AAF/I, II, III and IV), *aatA* (which corresponds to CVD432 fragment) encoding an outer-membrane protein TolC, which is a component of the *aat*-encoded ABC transporter involved in secretion of dispersin [1]. Dispersin is encoded by *aap* and allows dispersal of EAEC in the intestinal mucosa in humans [8]. In addition, a serine protease autotransporter toxin (Pet), which is able to cleave the host cell protein spectrin, and the lipopolysaccharide modulating *shf*-locus have been described [7], [9]. The final plasmid-encoded factor associated with virulence is AggR, the major regulator of EAEC virulence that controls expression of both plasmid- and chromosomally-encoded factors [10]. Chromosomally-encoded virulence factors identified for EAEC strains include the *Shigella* (*she*) and *Yersinia* pathogenicity islands, containing enterotoxins, mucinase genes, and iron acquisition genes [11]. In addition, adhesins, toxins and several other factors delivered by a type V secretion system (T5SS) or type VI secretion system (T6SS) have been implicated in pathogenesis [9], [10], [12], [13], [14]. Importantly, none of these factors has been consistently associated with EAEC-mediated disease and none of these factors are found in all EAEC strains.

In addition to the diversity of virulence factors, EAEC strains show substantial heterogeneity in virulence: EAEC strains are often isolated from asymptomatic individuals and in trials where healthy volunteers were fed EAEC strains 042, 17-2, JM221 and 34b, only EAEC 042 elicited diarrhoea [15]. Furthermore, like ETEC [16], [17], EAEC strains are genetically diverse and are found across all of the *E. coli* phylogenetic groups A, B1, B2 and D [18], [19], [20]. The heterogeneity of virulence factors, the genetic diversity of EAEC strains and the variation in clinical outcome following infection are major confounding factors in understanding EAEC and no correlation between pathogenicity in humans and specific genotype or phenotype has yet been possible [15], [21], [22], [23], [24].

In this study, we wished to determine if specific genotypic or phenotypic traits could be correlated with EAEC disease. Here we characterised 24 strains isolated during a case-control study of acute diarrhoea among children 0–24 months in Brazil [25]. Using several models of pathogenesis we were unable to distinguish between EAEC strains from controls or individuals with illness. Nevertheless, associations between the phylogroup of individual isolates and the risk of disease in humans was observed such that EAEC strains from phylogroup D are strongly associated with disease whereas strains from phylogroup A are more associated with asymptomatic controls.

## Results

### Genetic Profile of EAEC Clinical and Asymptomatic Cases Strains

The EAEC strains used in this study were previously identified from a large screen of 150 children aged less than two years old with acute diarrhoea and 50 age-matched healthy controls [25]. This study identified 990 *E. coli* isolates from stool samples. Fifteen of these isolates from children with clinical symptoms and nine from asymptomatic children were defined as EAEC by a positive test with the CVD432 probe and the ability to form an aggregative pattern of adherence on HEp-2 cells [6], [25]. The 24 EAEC strains belonged to a wide range of O-antigen and H-antigen serotypes (Table 1). Examination of the association between serotype and the source of the strain revealed no significant correlation between serotype and case or control status [25].

**Table 1:** Genetic attributes of EAEC strains.

Strains	Phylogenetic Group	Serotype		Virulence factors detected			
			Main EAEC markers	Aggregative adherence fimbriae	Iron uptake genes	T5SS-related genes	T6SS-related genes
**Isolates from Asymptomatic controls**							
** C2/4**	A	O153:H2	*aggR, aap*	*agg3A*	*fyuA, irp2*	*pic*	–
** C21/2**	A	O9:H10	*aap, astA*	–	*fyuA, irp2*	*pet,pic*,*sep*A	*sci*N/G/D
** C29/2**	A	O9:H10	*aggR, aap, astA*	–	*fyuA, irp2*	*pet,pic*,*sep*A	*sci*N/D
** C33/2**	A	O3:H2	*aggR, aap, astA*	*aggA*	*fyuA, irp2*	*pet,pic*,*sat*	*sci*N/D/*vgr*
** C46/3**	A	ONT:H30	*aap*	–	–	*pic*	–
** C44/5**	D	ONT:H23	*aap, astA*	–	*fyuA, irp2*	–	*sci*D
** C43/1**	B1	O152:H8	–	–	*fyuA, irp2*	*pet,pic*	–
** C52/2**	B1	ONT:H27	–	*aggA*	*fyuA, irp2*	*pic*	–
** C55/4**	B1	ONT:H10	*aggR, aap*	–	*fyuA, irp2*	*pet,pic,sat*	–
**Isolates from clinical cases**							
** H9/3**	A	O113:H12	*aggR, aap*	–	*fyuA, irp2*	*pet*	–
** H19/2**	A	ONT:H16	*aggR, aap, astA*	–	*fyuA, irp2*	*pet,pic*	–
** H63/2**	A	ONT:H^−^	*aggR, aap*	–	*fyuA, irp2*	*pic*	–
** H136/4**	A	O3:H2	*aggR, aap, astA*	*aggA*	*fyuA, irp2*	*pet,pic*	*sci*N/G/D *vgr*
** I49/3**	A	O117:H27	*aggR, aap, astA*	*agg3A*	*irp2*	*pet,pic*,*sep*A	*sci*N/G/D *vgr*
** H42/1**	D	O112:H21	*aggR, aap*	–	*fyuA, irp2*	*pet,pic*	–
** H80/3**	D	O119:H17	–	–	*fyuA, irp2*	*pic*	*sciD*
** H92/3**	D	O86:H18	*aggR, aap*	*aggA*	*fyuA, irp2*	*pic*	*sci*N/G/D *vgr*
** H102/1**	D	O86:H11	*aggR, aap*	*agg3A*	*fyuA, irp2*	–	–
** I18/2**	D	O86:H11	*aggR, aap*	–	*fyuA, irp2*	–	–
** I29/3**	D	ONT:H11	*aggR, aap*	–	*fyuA, irp2*	*pic*, *sep*A	*sci*N/G/D
** I34/4**	D	O111:H21	*aggR, aap, astA*	*agg3A*	*fyuA, irp2*	*pet,pic*,*sep*A	–
** H153/5**	B1	ONT:H21	–	–	–	–	–
** H1/4**	B2	ONT: H17	*aggR, aap, astA*	*aggA*	*fyuA, irp2*	*pet,pic,sat*	*sci*G/D
** H149/5**	B2	O5:H11	*astA*	–	*fyuA, irp2*	–	–
**Isolates tested in volunteer studies**							
**17-2**	**A**	O3:H2	*agg*R *aap*,	*aggA*	*irp*2	–	–
**JM221**	A	O92:H33	*agg*R, *aap,she*	*aggA*	–	–	–
**34b**	B1	ONT:H11	–	–	*irp2*	–	–
**042**	D	O44:H18	*agg*R, *aap*, *astA, she*	*aafA*	*irp*2	*pet, pic*	*sci*N/G/D *vgr*

In this study, to determine if EAEC virulence factors were more associated with strains from cases than from controls we tested each strain by PCR for the presence of 17 genes previously associated with EAEC virulence including the virulence regulator *aggR,* fimbrial genes, iron uptake genes, genes encoding Type 6 secretion system (T6SS)-related proteins and genes encoding autotransporters of the SPATE subfamily (Table 1) [26]. The previously characterised EAEC strains 042, 17-2, JM221 and 34b were included as controls. The gene encoding the AAF/I fimbria (*aggA*) was the most frequently identified pilin gene (25%) followed by AAF/III (*agg3A*; 11%) and AAF/II (*aafA;* 4%); the remaining strains (57%) were negative for known AAF fimbrial variants. For the remaining putative virulence factors, the irp2 gene (89%) was the most frequently detected gene followed by *aap* (79%), *fyuA* (75%), *aggR* (68%), *pic* (68%), *pet* (46%), *sciD* (40%), *astA* (36%), *sciN* (29%), *sciG* (25%) and *vgr* (18%). However, in no case was a specific virulence factor, or combination of virulence factors, more or less associated with diarrhoeal illness.

**Figure 1:**
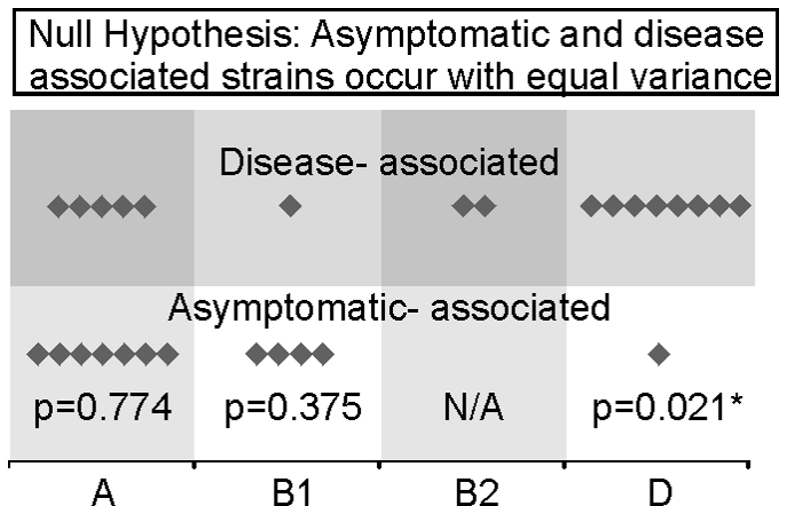
Distribution of asymptomatic and disease-associated EAEC strains across the four ECOR phylogroups. Strains from phylogroup A (12 strains) and B1 (5 strains) are equally distributed between the two groups. EAEC strains for phylogroup D (9 strains) are not equally distributed being highly associated with cases. There were not enough strains from phylogroup B2 (2 strains) for statistical analysis.

Previous investigations suggested commensal *E. coli* strains were more commonly associated with a specific evolutionary lineage of *E. coli*, namely the A phylogroup [27]. A previously described triplex PCR [28] was used to identify to which phylogenetic group each EAEC strain belonged (Table 1). This screening revealed the EAEC strains were distributed among all four A, B1, B2 and D phylogenetic groups, with a higher prevalence of A and D groups, representing 79% of all the isolated strains analysed. Using the null hypothesis that isolates from each phylogroup have an equal chance of being isolated from an asymptomatic or clinical (disease-associated) source, EAEC strains from phylogroup A and B1 fell within normal variation (Fig. 1). However, EAEC strains from phylogroup D were significantly (p = 0.021) outside normal variation and were more associated with disease than case controls (Fig. 1). There were not enough strains from phylogroup B2 to perform the statistical analysis.

### EAEC Strains Display Heterogeneous Levels of Adherence and Invasion in vitro

A critical first step in bacterial pathogenesis is the ability to adhere and invade human cells. All EAEC strains were tested for the pattern of adherence to Caco-2 and HT29 human cells. Some EAEC strains presented a differential pattern of adherence depending on the type of culture cells used (data not shown). However, all strains tested adhered in an aggregative adherence (AA) pattern to one cell type and most strains adhered in an AA pattern for both cell types. Importantly, no mutual correlation between adherence patterns or the level of adherence was found for either clinical or asymptomatic isolates. Therefore adherence to these cell lines is not a selective feature correlating with disease-associated isolates.

**Figure 2:**
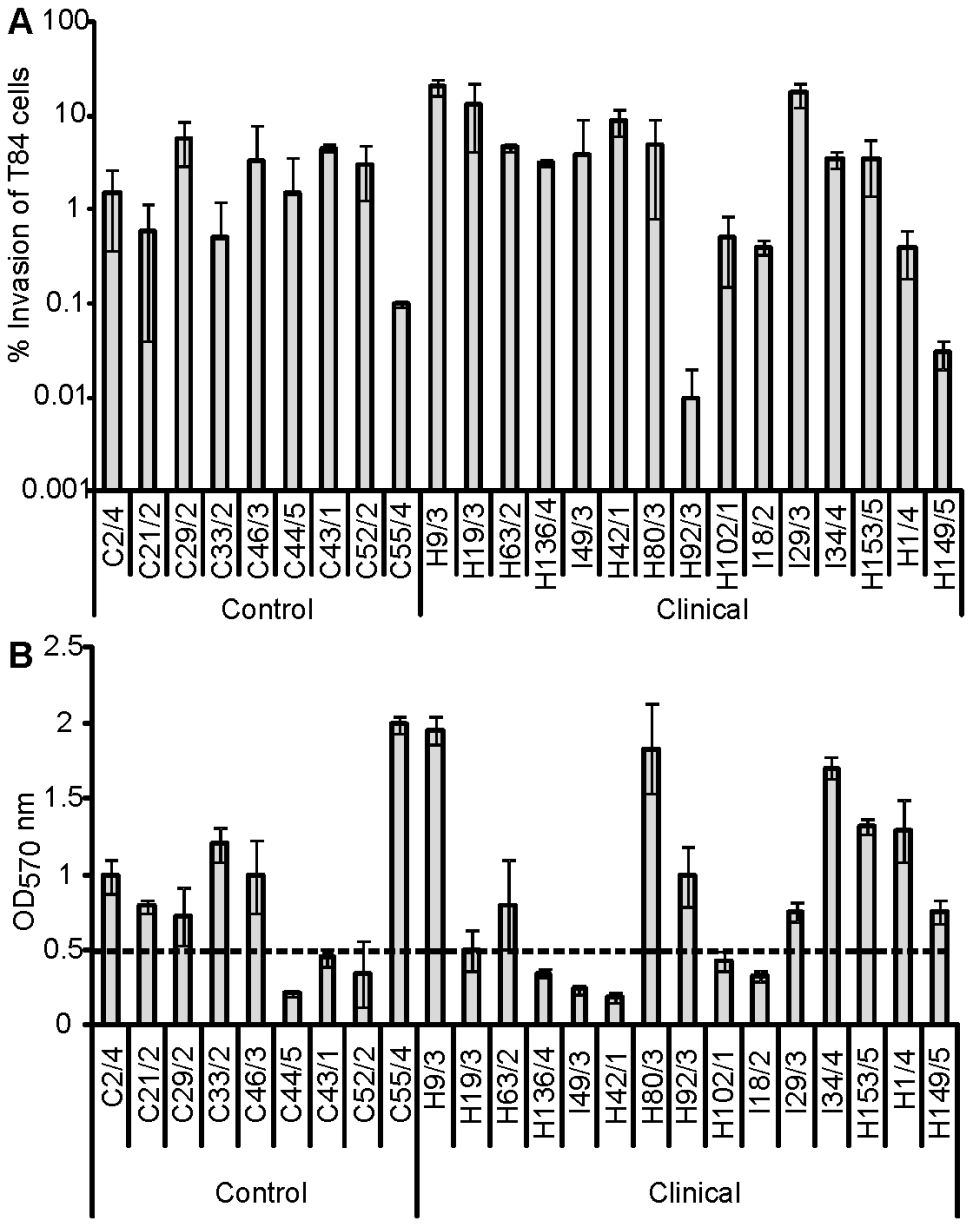
Phenotypic characterisation of EAEC strains. (A) Invasion levels showed by EAEC clinical and control strains after 3 h co-incubation with T84 cultured cells. No statistically significant difference between case or control strains was detected for the invasion assays. (B) Biofilm production on polystyrene determined semi-quantitatively according to OD_570_ values of strains screened for biofilm production. High biofilm production was defined when OD_570_ was higher/equal than 0.5.

Previous investigations revealed some EAEC strains possessed the ability to invade cells [29]. Therefore, the ability of the EAEC strains to invade T84 cell monolayers was quantified using a standard antibiotic protection assay. Most strains showed internalisation rates compatible with invasive strains [29]. However, levels of invasion varied greatly between EAEC strains from a low of 0.03% to a high of 20% (Fig. 2A). No correlation was found between internalisation rates and case or control origin of the strain; many isolates from controls had invasion rates similar to strains derived from symptomatic children (Fig. 2A). Therefore, the capacity to invade T84 cells was not predictive of the origin of these clinical isolates.

### EAEC Strains Display Heterogeneous Levels of Biofilm Formation

EAEC strains have previously been reported to have the capacity to establish biofilms [30]. Indeed, biofilm formation is thought to be an important step in colonisation and persistence of EAEC infection during human disease. Therefore, the ability of the EAEC strains to form a biofilm was tested. The ability to produce biofilm was assessed semi-quantitatively using the standard crystal violet-polystyrene microtitre plate assay described previously [30]. Sixteen (67%) strains demonstrated a high level of biofilm by this method. However, no significant difference in the ability to produce a biofilm was observed when strains from the symptomatic cohort were compared with strains from the control cases (Fig. 2B).

### EAEC Infection is Lethal in the *Caenorhabditis elegans* Model of Infection

**Figure 3:**
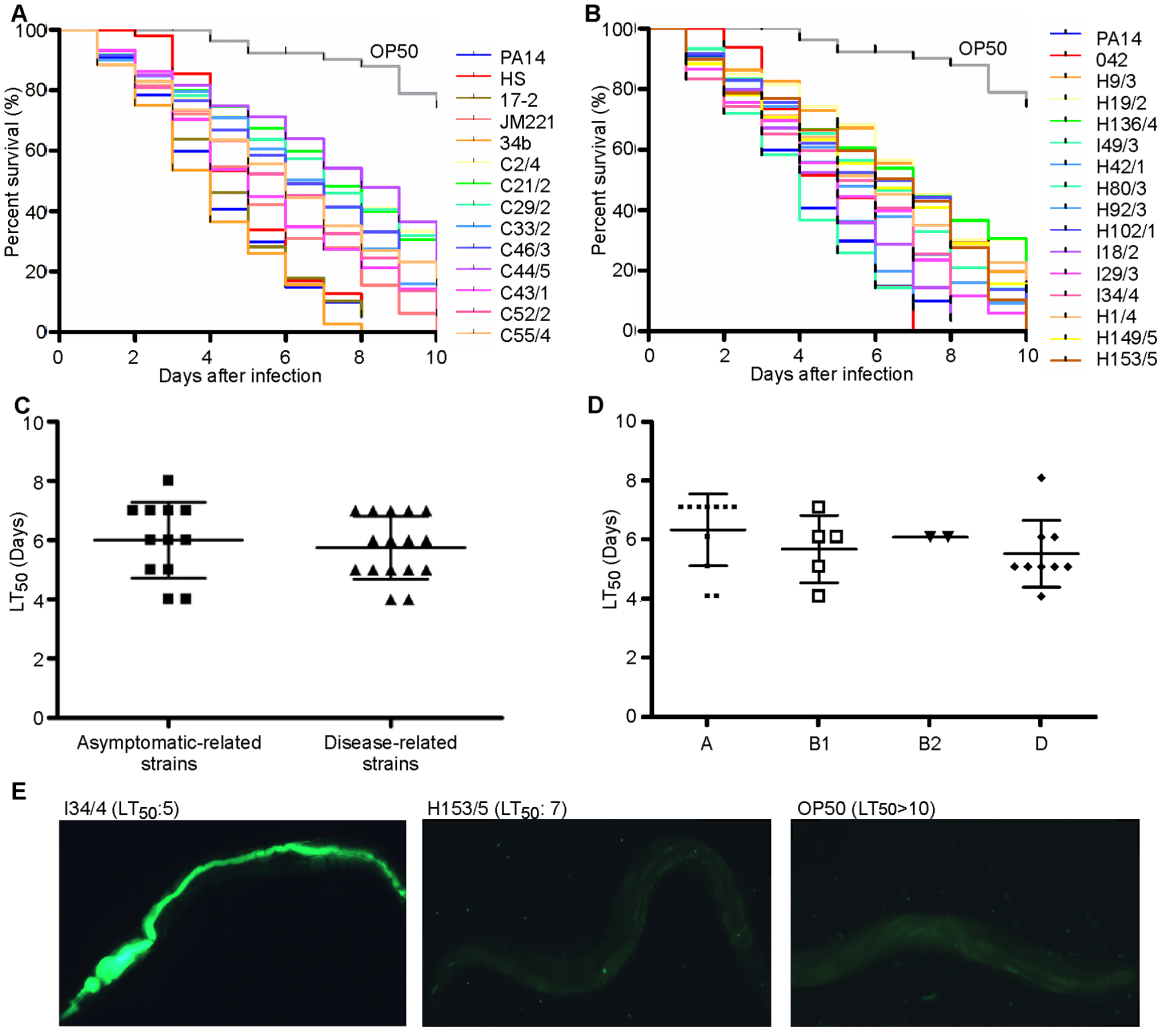
Rates of death in *C*.*elegans* slow killing assays. Nematode slow-kill virulence assays on NGM agar. The ability of each EAEC strain either associated with (A) asymptomatic or (B) clinical disease to kill the nematode worm *C. elegans* was examined over a 10 day period. The LT_50_ (time for half of the worms to die) was calculated for each experiment. The *E. coli* strain OP50, a commonly used as a food source for C. elegans, was used as a negative control (LT_50_>10). The opportunistic pathogen *P. aeruginosa* PA14 (LT_50_ = 4), which was previously shown to kill *C. elegans*, was included as a positive control. (C) Comparison between the LT_50_ exhibited by asymptomatic strains and clinical strains showed no statistically significant difference. D) Comparison of the LT_50_ of strains belonging to different phylogenetic groups showed that although phylogroup D showed the fastest killing rates, compared to strains belonging to groups A, B1 and B2 this was not statistically significant. (E) L4 nematodes were fed with EAEC clinical strains and negative control strain OP50 containing the pJB42-GFP expressing plasmid. After 72 h, worms were taken for imaging. Persistent colonisation of the worm intestine was found for strains with an LT_50_ of 4 or 5 (such as I34/4) in contrast with strains with an LT_50_ of 7 or more such as H153/5. Nematodes fed strain OP50 were not colonised. Magnification is at 100×.

Previous investigations suggested that the *C. elegans* model of infection could be used to study EAEC virulence [31], [32]. We hypothesised that strains from asymptomatic controls would be less virulent in this assay than strains harvested from patients with diarrhoea. Individual EAEC strains were fed to nematodes and the survival of the worms was measured for 10 days. All EAEC strains tested killed the worms faster than the negative control *E. coli* OP50, which is the normal food source for nematode growth in the laboratory (*P*<0.0001). However, there was a wide range of killing rates with the number of days it took to reduce the nematodes alive to 50% (LT_50_) varying between 4 and 8 days (Fig. 3A, B). Results showed that EAEC strains from asymptomatic children and EAEC strains isolated from children with diarrhoea were able to kill nematodes quickly. The average LT_50_ of the clinical isolates was lower (5.75±0.27) than asymptomatic isolates (6.000±0.37) but differences between the two groups were not statistically significant (*P* = 0.58) (Fig. 3C). These results agreed with results for the prototypical strains of EAEC with rates of killing similar between strains that failed to cause diarrhoea in volunteers (17-2, JM221 and 34b) and EAEC 042 that can elicit diarrhoea in humans. Importantly, these rates were similar to that observed for the human commensal strain *E. coli* HS (Fig. 3A).

Previous reports have suggested a link between phylogeny, virulence genes, origin of EAEC isolates and pathogenicity in humans [25], [31]. As we observed that phylogroup D strains were more associated with clinical cases than with asymptomatic controls we analysed whether phylogroup origin had an impact on the ability of a strain to kill *C. elegans*. These investigations revealed that strains from group A presented a slowest average killing (LT_50_: 6.25) compared to groups B1 (LT_50_: 5.6) and B2 (LT_50_: 6) and strains from group D presented the fastest average killing (LT_50_: 5.44) (Fig. 3D). However, the difference in the rate of *C. elegans* killing between phylogroups A and D was not statistically significant (*P* = 0.1382).

We next sought to establish if there was a relationship between the presence of particular virulence factors, the ability to biofilm formation and the ability to adhere to and invade cells *in vitro* and the ability of EAEC strains to kill *C. elegans*. Statistical analyses revealed no correlation between any of these factors and LT_50_ measured for *C. elegans* killing. However, we have previously established that other *E. coli* strains kill *C. elegans* by persistent colonisation of the nematode intestine [33]. To determine whether differences in nematode killing were due to the capacity of strains to colonise and form a biofilm in the nematode gut, *C. elegans* was fed on GFP-labelled EAEC strains and OP50. After three days of infection, GFP-expressing bacteria were visualised within the nematode gut through fluorescence microscopy. Interestingly, EAEC strains with a LT_50_≤5 days could be readily visualised along the entire length of the nematode gut (Fig. 3E). In contrast, strains with a longer LT_50_ had less evident colonisation after three days (Fig. 3E) and resembled worms fed with OP50 (Fig. 3E). Importantly, colonisation of the *C. elegans* intestine could be observed for strains from both asymptomatic and diseased individuals indicating that virulence in *C. elegans* is not an indicator of virulence in humans.

### The *C. elegans* Model can Identify Known Virulence Factors of EAEC 042

**Figure 4:**
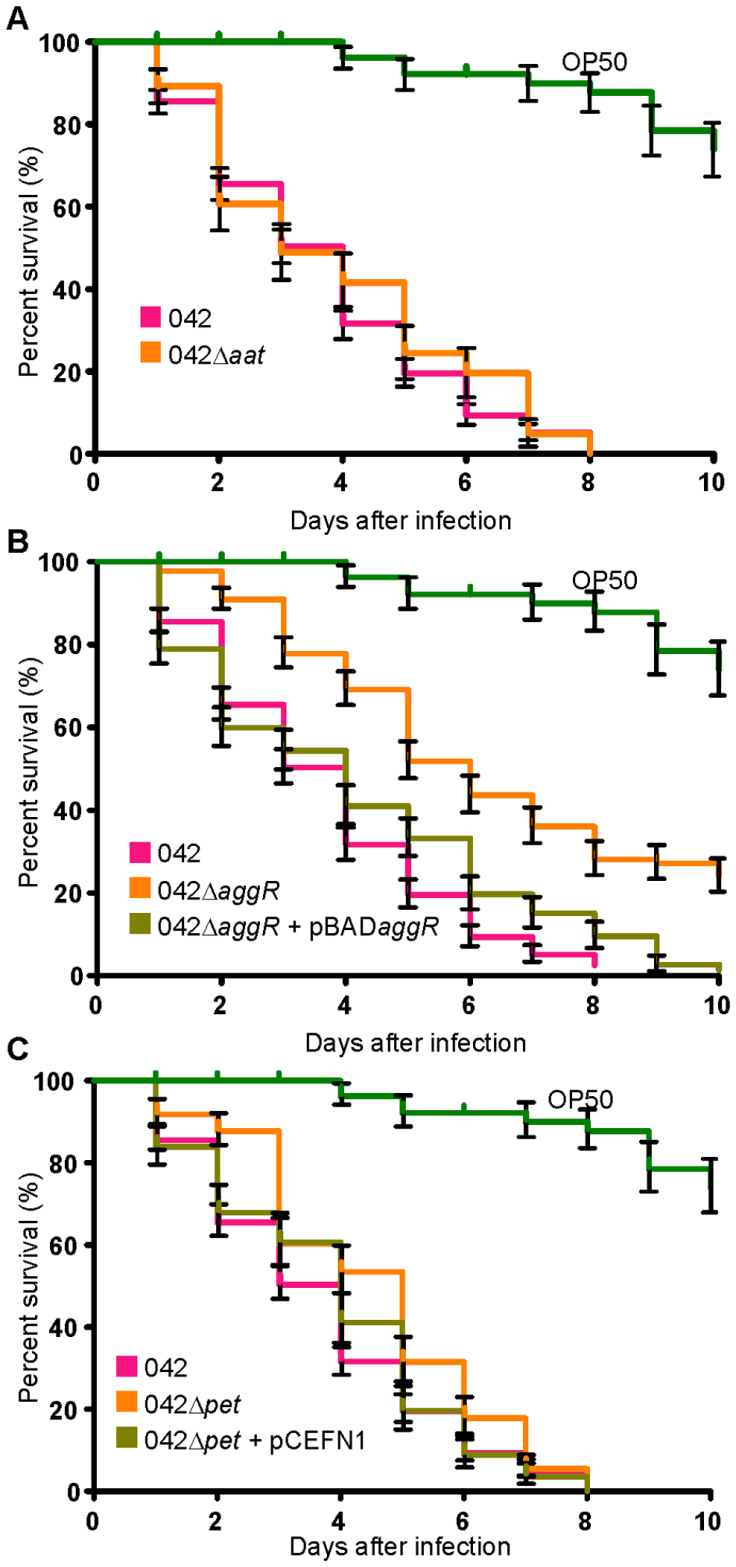
Deleting virulence factors from EAEC 042 slows *C.* *elegans* killing. Nematode slow-kill virulence assays on NGM agar. The ability of EAEC 042, (A) 042 Δ*aat,* (B) 042 Δ*aggR,* 042 Δ*aggR*+pBAD*aggR*, (C) 042 Δ*pet* and 042 Δ*pet*+pCEFN1 to kill the nematode worm *C. elegans* was examined over a 10 day period. Error bars represent +/− standard error. The 042 Δ*aggR* (LT_50_: 5.2, *P*<0.001) and 042 Δ*pet* (LT_50_: 4.29, *P*<0.05) strains had significantly higher LT_50_ than the 042 wildtype (LT_50_: 3.1). Complementation of these genes via pBAD*aggR* (LT_50_: 3.4) or pCEFN1 (LT_50_: 3.6) restored the LT_50_ to wildtype levels. The 042 Δ*aat* strain had an LT_50_ similar to wildtype (LT_50_: 2.9).

Although we could not find a correlation between the rate of nematode killing and presence of specific virulence genes or any phenotypic properties, such as biofilm formation and invasion ability, we hypothesised that the *C. elegans* model may be useful for identifying single virulence factors. To determine if this was the case, we compared the rate of killing between the human pathogenic EAEC strain 042 and a selection of EAEC 042 mutants lacking specific virulence factors. This analysis revealed that the LT_50_ of the isogenic mutants EAEC 042 Δ*aggR* (5.2+/−0.43) and EAEC 042 Δ*pet* (4.29+/−0.454) were significantly longer (*P*<0.001 and *P*<0.05 respectively) than that observed for the parental EAEC strain 042 (3.1+/−0.346). Complementation of the two mutants with plasmids containing either *aggR* (pBAD*aggR*) or *pet* (pCEFN1) restored the LT_50_ to wildtype levels (3.4 and 3.6 respectively). In contrast, no difference in nematode killing was seen when the *aat* operon was deleted (Fig. 4A). These results suggest that the *C. elegans* model may be useful for identifying and studying specific virulence factors from EAEC strains.

## Discussion

One of the major challenges to understanding EAEC pathogenicity and epidemiology is the high level of heterogeneity observed amongst EAEC strains. Heterogeneity has been observed in the repertoire of virulence factors present in strains [21], [22], in genetic lineages from which EAEC have evolved, and in the diseases state of the host from which EAEC strains are isolated. Many investigations have sought to correlate the presence of EAEC virulence factors, or sets of virulence factors, with disease [21], [23], [24], [28]. Whilst the results of these studies often do not agree with each other on the frequency of specific virulence factors within the EAEC population, most have the same outcome as this present study viz. no single virulence factor examined to date has a significant correlation with disease and currently no simple genetic method can be used to distinguish pathogenic EAEC strains from non-pathogenic strains [19], [23], [34], [35].

Although no clear association exists between the repertoire of virulence factors and the ability of EAEC to cause disease, the correlation between EAEC phenotypes and pathogenesis has remained enigmatic. Here we sought to determine if phenotypes derived from *in vitro* assays correlated with the ability of EAEC to cause disease. The standard assay to define EAEC is the adherence of bacterial strains to HEp-2 cells in a classic “stacked-brick” pattern of adherence [1]. We sought to determine if adherence to the more relevant colonic cells lines T84 and Caco-2 was indicative of EAEC association with a diseased state. Interestingly, we observed heterogeneity in the levels of adherence and in the pattern of adherence to these cell lines. However, these data did not correlate with isolates from either clinical or control subjects. Heterogeneity in the ability of EAEC strains to invade colonic cell lines has also been reported [29]. Therefore, we sought to determine if differences in invasion were reflective of EAEC strains associated with a diseased host. As with the adherence assays, we observed heterogeneity of the level of invasion but again no correlation with case or controls was found. The ability of EAEC to form biofilms is a long recognised feature of this pathotype, and indeed biofilm formation on human colonic tissue has been reported [30]. Furthermore, loss of biofilm promoting factors diminishes the ability of EAEC to colonise the mouse intestine [36], [37]. However, there was no significant difference in the ability of control EAEC strains to form biofilms when compared to EAEC isolates from symptomatic children. Thus, it appears that *in vitro* phenotypic assays do not appear to correlate with EAEC *in vivo* colonisation and pathogenesis.

Since the *in vitro* models could not distinguish between EAEC strains isolated from cases and controls we sought to determine if the *C. elegans* model of infection would be discriminating. Our results show that EAEC strains do vary in the rate they kill *C. elegans* with the number of days to reduce the initial nematode population to 50% (LT_50_) varying between 4 and 8. Although clinically isolated EAEC strains tended to kill the worms faster than strains from healthy controls, this was not found to be significant. Furthermore, it is worth noting that although the human pathogen EAEC 042 did kill the worms in the fastest time (LT_50_ = 4), the three EAEC strains (17-2, JM221 and 34b) that did not cause disease in human challenge studies [15] also had statistically similar rates of killing. Thus, it seems that fast killing *C. elegans* does not indicate pathogenicity in humans. Indeed, we have recently shown that killing of *C. elegans* by EAEC 042 occurs at a similar rate to the nonpathogenic human commensal strain *E. coli* HS and to the laboratory adapted *E. coli* strain K-12 (when lipopolysaccharide O-antigen production has been restored) [33]. In the same study we demonstrated that killing was the result of the colonisation and growth within the nematode gut causing distension and eventual rupture of the intestine, rather than associated with the release of toxins, a phenotype displayed by other bacterial pathogens [38], [39]. These data suggest that genes that promote survival in the nematode grinder, fast colonisation and growth are essential for pathogenesis of bacteria in this model and are not directly reflective of the ability to cause disease in humans.

While the *C. elegans* model does not appear suited to discriminating between pathogenic and non-pathogenic EAEC strains, our results suggest it may be useful in identifying virulence factors from pathogenic strains. Microbial genes from *Pseudomonas aeruginosa,* enterohemorrhagic *E. coli* and *Salmonella spp.,* known to be important for virulence in mammalian models, have been shown to be required for full virulence in nematodes [39], [40], [41]. In this regard, the *C. elegans* model may be a simpler and more ethical substitute to murine and rabbit models of infection when investigating virulence factors of newly isolated EAEC strains. However, it is interesting to note that while inactivation of *aggR* led to a decrease in EAEC 042 lethality for *C. elegans* and abrogation of Aap surface expression (through an *aat* deletion) had no effect on nematode killing the opposite was true in a murine model of EAEC 042 colonisation: an isogenic *aggR* mutant was unaffected in colonisation of the murine gut whereas an isogenic *aap* mutant failed to establish a persistent colonisation [8], [42]. These data clearly indicate that establishing an appropriate model of pathogenesis is essential to understanding which strains of EAEC elicit disease and elucidating the mechanism by which they induce diarrhea.

As mentioned previously, EAEC strains display heterogeneity in their genetic lineage; like ETEC they have a polyphyletic origin and can be found across the breadth of the *E. coli* phylogeny [17], [34], [35]. Escobar-Paramo *et al.* [43] suggested that certain genetic backgrounds are required for acquisition and expression of virulence factors, thus certain bacterial lineages would be more related to disease [35], [43]. Previously, researchers characterising EAEC isolates from childhood diarrhoea in Nigeria, showed the predominance of phylogroup D (including EAEC 042) and the ST10 complex lineage comprising phylogroup A among the isolates and suggesting that these pathotypes should deserve further attention [35]. Our data also suggests that EAEC strains from phylogroup D may be much more likely to cause disease in humans than EAEC strains from other phylogroups. While we recorded no significant association between the A phylogroup and isolates from asymptomatic individuals; it is notable that the majority of commensal *E. coli* are associated with phylogroup A and two of the strains which failed to cause disease in human volunteers also belonged to phylogroup A [15]. However, it is worth recalling many studies have demonstrated the presence of EAEC strains in healthy controls (ca. 2%) and that the rate of recovery of EAEC in symptomatic individuals is approximately 3-fold higher [44], [45]. Therefore, a simplistic hypothesis would suggest that approximately one third of the strains isolated from symptomatic individuals are not associated with disease but simply part of the resident commensal flora. Notably 5/15 strains isolated from patients with disease belonged to phylogroup A. If these are simply part of the normal resident flora then the association of phylogroup D with disease would increase significantly.

In conclusion, we have demonstrated that none of the models tested here adequately distinguishes between EAEC isolates from asymptomatic and symptomatic individuals. In contrast, an association between phylogroup D and EAEC-mediated disease was observed. These data suggest further efforts to distinguish between commensal and pathogenic EAEC should focus on genetically characterising a large cohort of EAEC strains from different geographic regions to ascertain whether the observations made here hold true in other clinical settings.

## Materials and Methods

### Strains, Plasmids and Primers

Bacterial strains used this study included the EAEC strains 042, 34b, 17-2 and JM221 and 24 clinical and control cases EAEC isolates of our collection and are detailed in Table 1. These previously described EAEC isolates {Rosa, 1998 #139} were the sole identifiable pathogen taken from children aged less than 2 years either presenting acute diarrhoea or no diarrhoeagenic symptoms (Table 1). In addition to EAEC strains, all other strains, plasmids and primers used in the study are found in Tables S1 and S2. All strains were routinely stored at −70°C in lysogeny broth (LB) (Sigma) supplemented with 15% glycerol.

### Adherence Assays

EAEC strains were analysed for their pattern of adherence to cultured human colon carcinoma cells as previously described [25]. Briefly, semiconfluent HT-29 and confluent Caco-2 cultured epithelial cell monolayers were grown in 24-well tissue culture plates over glass slides, in Dulbecco's modified Eagle's medium (DMEM, Sigma) with 10% foetal calf serum (FCS, Gibco-BRL, USA) at 37°C in 5% CO_2_. Prior to infection, the cell culture medium in the plates was replaced with minimal essential medium supplemented with 2% FCS and 1% D-mannose without antibiotics. *E. coli* isolates to be tested were grown overnight in Trypticase soy broth (Oxoid) at 37°C, without shaking. 35 µl of overnight bacterial culture adjusted to OD_600_ = 2 were inoculated over the cells monolayers, and the co-culture was incubated at 37°C in 5% CO_2_ during a total of 3 h. Slides were washed twice with phosphate-buffered saline (PBS), fixed with 100% methanol, and stained with Giemsa (1∶20 v/v in PBS). Control strains EAEC 042 and *E. coli* HB101 or DH5α were included in each assay. Each *E. coli* strain was tested in triplicate.

### Invasion Assays

The protocol was performed as previously described [29]. Briefly, T84 cells monolayers were seeded in 24-well tissue culture plates and incubated for 2–3 days in a 1∶1 mixture of Ham F12 and DMEM supplemented with 10% FCS, 5 mM glutamine and antibiotics. Cell monolayers were washed twice with PBS (pH 7.2) and the cell culture medium was then replaced with minimal essential medium supplemented with 2% of FCS and 1% D-mannose without antibiotics. Bacteria were grown overnight in Trypticase soy broth (Oxoid) at 37°C, without shaking. Each monolayer was infected with 100 µl of the cell culture, containing 1×10^8^ bacteria. After a total of 3 h incubation period at 37°C with 5% CO_2_, infected monolayers were washed twice with PBS. For measurement of invasion and to evaluate the number of intracellular bacteria, cell culture medium with 250 µg/ml amikacin was used in each slot, added to kill extracellular bacteria. After incubation for an additional hour, monolayers were washed two times with PBS, and 1 ml of 1% Triton X-100 in deionised water was placed in each well for 5 min to lyse the eukaryotic cells. This concentration of Triton X-100 did not affect bacterial viability for at least 30 min. Samples were removed, diluted, and plated onto Trypticase soy agar (Oxoid) plates to determine the number of CFU recovered from the lysed monolayers. Invasion levels were expressed as the percentage of the original inoculum resisting treatment with amikacin. To determine the total number of cell-associated bacteria corresponding to adherent and intracellular bacteria, eukaryotic cells were lysed after the 3 h infection period and the bacteria were quantified. All assays were performed at least three times in independent experiments.

### Biofilm Semi-quantitative Assays

All 24 EAEC clinical and control cases strains were tested for their capacity of adhering to glass coverslips to evaluate biofilm formation as previously described [30]. Bacteria were cultured at 37°C in Trypticase soy agar (Oxoid) with shaking. Aliquotes of 5 µL of each culture were transferred to 200 µL of Dulbecco modified Eagle’s medium (DMEM, Sigma), containing 0.45% glucose, previously distributed into each well of a 96-well polystyrene microtitre plate. Plate containing the bacterial cultures was placed into an incubator at 37°C, for 18 h. After the incubation period, medium was removed and each well was washed with Milli-Q water three times and stained with 50 µL of 0.5% crystal violet (Laplast Labor), for 5 min. After this period, wells were washed three times followed by the addition of 200 µL of ethanol to each well for 30 min. Aliquots of 150 µL were transferred to a new polystyrene plate and the optical density was measured at 570 nm in a spectrophotometer. Assays were performed in triplicate.

### *C. elegans* Infection Model

*C. elegans* assays were performed as previously described [33]. Briefly, EAEC strains were grown in LB and plated onto Nematode Growth Media (NGM) agar, for 48 h, at 25°C. After the period, twenty N2 nematodes at L4 stage were transferred to each assay plate using a platinum wire. Animals were transferred to new inoculated plates every two days in order to allow the distinction of subsequent generations. Data from the assays were analysed by Kaplan-Meier statistical method using statistical analysis program GraphPad Prism version 5.00 for Windows, GraphPad Software, San Diego, California, USA (www.graphpad.com). The assays were conducted in triplicates.

### Colonisation Assay

Synchronised nematodes at stage L4 were subjected to infection by EAEC and control strains electroporated with pJB42 [33], a GFP expressing plasmid. Strains were grown in NGM plates supplemented with ampicillin (100 µL/mL) or kanamicin (50 µg/mL), at 25°C. After 72 h of infection, nematodes were removed and placed in 200 µl of M9 minimal medium buffer previously disposed in a NGM plate and washed to remove adherent bacteria from nematode surface. Nematodes were washed three times in M9 buffer and then re-suspended in 100 µL of 4% NFP solution. Re-suspensions were transferred to 1% agarose pads on glass microscope slides. Colonisation of nematodes intestinal tract was visualised using an upright confocal microscope (Leica).

### Statistics

All experiments were repeated in triplicate. Means from each experiment of biofilm production and internalisation assays were analysed by Student’s *t*-test using Prisma statistical software. Variance was determined using a one-sample binomial test using the SPSS software as well as a Fisher’s Exact test. Statistically results were considered significant when *P*≤0.05. The survival curves of *C. elegans* interacting with different EAEC strains were compared with the survival curve of *C. elegans* fed with EAEC 042. The log-rank test was used to compare the survival differences for statistical significance.

## Supporting Information

Table S1 **Strains and plasmids used in this study.**
(DOCX)

Table S2 **Primers used in PCR reactions.**
(DOCX)
