# Supplementary material for: Genotypic and Phenotypic Characterisation of Enteroaggregative Escherichia coli from Children in Rio de Janeiro, Brazil
Source: PLoS One. 2013 Jul 30;8(7):e69971. doi: 10.1371/journal.pone.0069971 (PMC3728331; doi:10.1371/journal.pone.0069971)
Supplement: Table S1 — Strains and plasmids used in this study. (DOCX) [file pone.0069971.s001.docx]

**Table S1. Strains and plasmids used in this study**

| Strain | Characteristics | Reference |
| --- | --- | --- |
| *Pseudomonas aeruginosa* PA14 | Clinical *Pseudomonas* isolate | [[1](#_ENREF_1)] |
| *Escherichia coli* OP50 | *E. coli* B strain | [[2](#_ENREF_2)] |
| *Escherichia coli* HS | Wildtype commensal *E. coli* strain | [[3](#_ENREF_3)] |
| *Escherichia coli* 042 | Wildtype EAEC of proven pathogenicity | [[4](#_ENREF_4)] |
| *Escherichia coli* JM221 | Wildtype EAEC proven not to cause disease | [[4](#_ENREF_4)] |
| *Escherichia coli* 34b | Wildtype EAEC proven not to cause disease | [[4](#_ENREF_4)] |
| *Escherichia coli* 17-2 | Wildtype EAEC proven not to cause disease | [[4](#_ENREF_4)] |
| 042Δ*aggR* | 042 harbouring pJP5603 integrated into the integrated into the gene *aggR* (Km) | [[5](#_ENREF_5)] |
| 042Δ*pet* | 042 harbouring pJP5603 integrated into the integrated into the gene *pet* (Km) | [[6](#_ENREF_6)] |
| 042Δ*aat* | 042 harbouring pJP5603 integrated into the *aat* operon (Km) | [[7](#_ENREF_7)] |
|  |  |  |
| Plasmids |  |  |
| pJP5603 | 3.1-kb R6K suicide plasmid (Km) | [[8](#_ENREF_8)] |
| pJB42 | GFP expressing vector constructed from pDOC-C | [[9](#_ENREF_9)] |
| pBAD*aggR* | AggR cloned into pBAD30 for expression with arabinose (Ap) | [[10](#_ENREF_10)] |
| pCEFN1 | 3.9-kb PCR-derived fragment expressing Pet protein cloned into pSPORT1 (Ap) | [[11](#_ENREF_11)] |

**References from supplementary information**

1. Rahme LG, Stevens EJ, Wolfort SF, Shao J, Tompkins RG, et al. (1995) Common virulence factors for bacterial pathogenicity in plants and animals. Science 268: 1899-1902.

2. Brenner S (1974) The genetics of Caenorhabditis elegans. Genetics 77: 71-94.

3. Levine MM, Bergquist EJ, Nalin DR, Waterman DH, Hornick RB, et al. (1978) Escherichia coli strains that cause diarrhoea but do not produce heat-labile or heat-stable enterotoxins and are non-invasive. Lancet 1: 1119-1122.

4. Nataro JP, Deng Y, Cookson S, Cravioto A, Savarino SJ, et al. (1995) Heterogeneity of enteroaggregative Escherichia coli virulence demonstrated in volunteers. J Infect Dis 171: 465-468.

5. Sheikh J, Hicks S, Dall'Agnol M, Phillips AD, Nataro JP (2001) Roles for Fis and YafK in biofilm formation by enteroaggregative Escherichia coli. Mol Microbiol 41: 983-997.

6. Henderson IR, Hicks S, Navarro-Garcia F, Elias WP, Philips AD, et al. (1999) Involvement of the enteroaggregative Escherichia coli plasmid-encoded toxin in causing human intestinal damage. Infect Immun 67: 5338-5344.

7. Nishi J, Sheikh J, Mizuguchi K, Luisi B, Burland V, et al. (2003) The export of coat protein from enteroaggregative Escherichia coli by a specific ATP-binding cassette transporter system. J Biol Chem 278: 45680-45689.

8. Penfold RJ, Pemberton JM (1992) An improved suicide vector for construction of chromosomal insertion mutations in bacteria. Gene 118: 145-146.

9. Browning DF, Wells TJ, Franca FL, Morris FC, Sevastsyanovich YR, et al. (2013) Laboratory adapted Escherichia coli K-12 becomes a pathogen of Caenorhabditis elegans upon restoration of O antigen biosynthesis. Mol Microbiol.

10. Sheikh J, Czeczulin JR, Harrington S, Hicks S, Henderson IR, et al. (2002) A novel dispersin protein in enteroaggregative Escherichia coli. J Clin Invest 110: 1329-1337.

11. Eslava C, Navarro-Garcia F, Czeczulin JR, Henderson IR, Cravioto A, et al. (1998) Pet, an autotransporter enterotoxin from enteroaggregative Escherichia coli. Infect Immun 66: 3155-3163.

12. Baudry B, Savarino SJ, Vial P, Kaper JB, Levine MM (1990) A sensitive and specific DNA probe to identify enteroaggregative Escherichia coli, a recently discovered diarrheal pathogen. J Infect Dis 161: 1249-1251.

13. Nataro JP, Yikang D, Yingkang D, Walker K (1994) AggR, a transcriptional activator of aggregative adherence fimbria I expression in enteroaggregative Escherichia coli. J Bacteriol 176: 4691-4699.

14. Nataro JP, Deng Y, Maneval DR, German AL, Martin WC, et al. (1992) Aggregative adherence fimbriae I of enteroaggregative Escherichia coli mediate adherence to HEp-2 cells and hemagglutination of human erythrocytes. Infect Immun 60: 2297-2304.

15. Yamamoto T, Nakazawa M (1997) Detection and sequences of the enteroaggregative Escherichia coli heat-stable enterotoxin 1 gene in enterotoxigenic E. coli strains isolated from piglets and calves with diarrhea. J Clin Microbiol 35: 223-227.

16. Czeczulin JR, Balepur S, Hicks S, Phillips A, Hall R, et al. (1997) Aggregative adherence fimbria II, a second fimbrial antigen mediating aggregative adherence in enteroaggregative Escherichia coli. Infect Immun 65: 4135-4145.

17. Bernier C, Gounon P, Le Bouguenec C (2002) Identification of an aggregative adhesion fimbria (AAF) type III-encoding operon in enteroaggregative Escherichia coli as a sensitive probe for detecting the AAF-encoding operon family. Infect Immun 70: 4302-4311.

18. Schubert S, Rakin A, Karch H, Carniel E, Heesemann J (1998) Prevalence of the "high-pathogenicity island" of Yersinia species among Escherichia coli strains that are pathogenic to humans. Infect Immun 66: 480-485.

19. Restieri C, Garriss G, Locas MC, Dozois CM (2007) Autotransporter-encoding sequences are phylogenetically distributed among Escherichia coli clinical isolates and reference strains. Appl Environ Microbiol 73: 1553-1562.

20. Boisen N, Ruiz-Perez F, Scheutz F, Krogfelt KA, Nataro JP (2009) Short report: high prevalence of serine protease autotransporter cytotoxins among strains of enteroaggregative Escherichia coli. Am J Trop Med Hyg 80: 294-301.

21. Clermont O, Bonacorsi S, Bingen E (2000) Rapid and simple determination of the Escherichia coli phylogenetic group. Appl Environ Microbiol 66: 4555-4558.
