# Supplementary material for: Genotypic and Phenotypic Characterisation of Enteroaggregative Escherichia coli from Children in Rio de Janeiro, Brazil
Source: PLoS One. 2013 Jul 30;8(7):e69971. doi: 10.1371/journal.pone.0069971 (PMC3728331; doi:10.1371/journal.pone.0069971)
Supplement: Table S2 — Primers used in PCR reactions. (DOCX) [file pone.0069971.s002.docx]

**Table S2. Primers used in PCR reactions**

| **Primers** | **Sequences ( 5´→ 3´)** | **Target** | **Length (bp)** | **Reference** |
| --- | --- | --- | --- | --- |
| **Main EAEC markers** |  |  |  |  |
| CVD432  Continua | CTGGCGAAAGACTGTATCAT | pAA fragment | 630 | [[12](#_ENREF_12)] |
|  | CAATGTATAGAAATCCGCTGTT |  |  |  |
| AggR F | CTAATTGTACAATCGATGTA | Major regulon of EAEC virulence factors | 308 | [[13](#_ENREF_13)] |
| AggR R | ATGAAGTAATTCTTGAAT |  |  |  |
| Aap F | CTTTTCTGGCATCTTGGGT | Dispersin (*aap*) | 232 | [[14](#_ENREF_14)] |
| Aap R | GTAACAACCCCTTTGGAAGT |  |  |  |
| EAST F | CCATCAACACAGTATATCCGA | EAST1 heat-stable toxin (*astA*) | 111 | [[15](#_ENREF_15)] |
| EAST R | GGTCGCGAGTGACGGCTTTGT |  |  |  |
| **Aggregative adherence fimbriae** |  |  |  |  |
| AggA F | TTAGTCTTCTATCTAGGG | AAF/I fimbrial fragment (*aggA*) | 457 | [[14](#_ENREF_14)] |
| AggA R | AAATTAATTCCGGCATGG |  |  |  |
| AafA F | ACATGCATGCAAAAAATCAGAATGTTTGTT | AAF/II fimbrial fragment (*aafA*) | 550 | [[16](#_ENREF_16)] |
| AafA R | AAATTAATTCCGGCATGG |  |  |  |
| Agg-3A F | GTATCATTGCGAGTCTGGTATTCAG | AAF/III fimbrial fragment (*agg3A*) | 462 | [[17](#_ENREF_17)] |
| Agg-3A R | GGGCTGTTATAGAGTAACTTCCAG |  |  |  |
| **Iron uptake genes** |  |  |  |  |
| FyuA F | GCGACGGGAAGCGATTTA | Yersina bactin syderophore (*fyuA*) | 780 | [[18](#_ENREF_18)] |
| FyuA R | CGCAGTAGGCACGATGTTGTA |  |  |  |
| Irp2 F | AAGGATTCGCTGTTACCGGAC | Yersina bactin syderophore (*irp2*) | 280 | [[18](#_ENREF_18)] |
| Irp2 R | TCGT1CGGGCAGCGTTTCTTCT |  |  |  |
| **T5SS- related genes** |  |  |  |  |
| Pet F | GGCACAGAATAAAGGGGTGTTT | Serine-protease autotransporter /cytotoxic activity | 302 pb | [[19](#_ENREF_19)] |
| Pet R | CCTCTTGTTTCCACGACATAC |  |  |  |
| Pic F | ACTGGATCTTAAGGCTCAGGAT | Protein involved in colonization / mucinase | 572pb | [[19](#_ENREF_19)] |
| Pic R | GACTTAATGTCACTGTTCAGCG |  |  |  |
| SepA F | GCAGTGGAAATATGATGCGGC | *Shigella* extracellular protein A / enterotoxigenic | 794pb | [[19](#_ENREF_19)] |
| SepA R | TTGTTCAGATCGGAGAAGAACG |  |  |  |
| Sat F | TCAGAAGCTCAGCGAATCATTG | Secreted autotransporter toxin | 930 pb | [[20](#_ENREF_20)] |
| Sat R | CCATTATCACCAGTAAAACGCACC |  |  |  |
| EspP F | GTCCATGCAGGGACATGCCA | Extracellular plasmid encoded protease | 547bp | [[19](#_ENREF_19)] |
| EspP R | TCACATCAGCACCGTTCTCTAT |  |  |  |
| SigA F | CCGACTTCTCACTTTCTCCCG | Cytotoxic activity | 430bp | [[20](#_ENREF_20)] |
| SigA R | CCATCCAGCTGCATAGTGTTTG |  |  |  |
| **T6SS- related genes** |  |  |  |  |
| SciN F | TATCGCTGGTAAGGCTGGTT | Outer-membrane lipoprotein required for secretion of SciD protein | 579 | This work |
| SciN R | GCCAGTGAAATTGCCATAGA |  |  |  |
| Vgr F | CAGGTGATGCTGAGTGCTGT | Secreted protein/ efector function | 652 | This work |
| Vgr R | GGCGGATAGCTTCATCCATA |  |  |  |
| SciG F | ACGTCAGTTACCGGACAAGG | Secreted protein /cytotoxin | 971 | This work |
| SciG R | GTTGCTGCATCAGAAGGTCA |  |  |  |
| SciD F | TGGCAATTCCAGTTTATCTGTG | Secreted protein/ efector function (Hcp-like) | 465 | This work |
| SciD R | CGTCGGAATGAATGATGTTG |  |  |  |
| **Triplex PCR to identify phylogenetic group** |  |  |  |  |
| ChuA.1 | GACGAACCAACGGTCAGGAT | Protein involved in hemin transportation (*chuA*) | 279 | [[21](#_ENREF_21)] |
| ChuA.2 | TGCCGCCAGTACCAAAGACA |  |  |  |
| YjaA.1 | TGAAGTGTCAGGAGACGCTG | Gene found in *E. coli* K-12 (*yjaA*) | 211 | [[21](#_ENREF_21)] |
| YjaA.2 | ATGGAGAATGCGTTCCTCAAC |  |  |  |
| TspE4.C2.1 | GAGTAATGTCGGGGCATTCA | Esterase-lipase protein | 152 | [[21](#_ENREF_21)] |
| TspE4.C2.2 | CGCGCCAACAAAGTATTACG |  |  |  |

**References from supplementary information**

1. Rahme LG, Stevens EJ, Wolfort SF, Shao J, Tompkins RG, et al. (1995) Common virulence factors for bacterial pathogenicity in plants and animals. Science 268: 1899-1902.

2. Brenner S (1974) The genetics of Caenorhabditis elegans. Genetics 77: 71-94.

3. Levine MM, Bergquist EJ, Nalin DR, Waterman DH, Hornick RB, et al. (1978) Escherichia coli strains that cause diarrhoea but do not produce heat-labile or heat-stable enterotoxins and are non-invasive. Lancet 1: 1119-1122.

4. Nataro JP, Deng Y, Cookson S, Cravioto A, Savarino SJ, et al. (1995) Heterogeneity of enteroaggregative Escherichia coli virulence demonstrated in volunteers. J Infect Dis 171: 465-468.

5. Sheikh J, Hicks S, Dall'Agnol M, Phillips AD, Nataro JP (2001) Roles for Fis and YafK in biofilm formation by enteroaggregative Escherichia coli. Mol Microbiol 41: 983-997.

6. Henderson IR, Hicks S, Navarro-Garcia F, Elias WP, Philips AD, et al. (1999) Involvement of the enteroaggregative Escherichia coli plasmid-encoded toxin in causing human intestinal damage. Infect Immun 67: 5338-5344.

7. Nishi J, Sheikh J, Mizuguchi K, Luisi B, Burland V, et al. (2003) The export of coat protein from enteroaggregative Escherichia coli by a specific ATP-binding cassette transporter system. J Biol Chem 278: 45680-45689.

8. Penfold RJ, Pemberton JM (1992) An improved suicide vector for construction of chromosomal insertion mutations in bacteria. Gene 118: 145-146.

9. Browning DF, Wells TJ, Franca FL, Morris FC, Sevastsyanovich YR, et al. (2013) Laboratory adapted Escherichia coli K-12 becomes a pathogen of Caenorhabditis elegans upon restoration of O antigen biosynthesis. Mol Microbiol.

10. Sheikh J, Czeczulin JR, Harrington S, Hicks S, Henderson IR, et al. (2002) A novel dispersin protein in enteroaggregative Escherichia coli. J Clin Invest 110: 1329-1337.

11. Eslava C, Navarro-Garcia F, Czeczulin JR, Henderson IR, Cravioto A, et al. (1998) Pet, an autotransporter enterotoxin from enteroaggregative Escherichia coli. Infect Immun 66: 3155-3163.

12. Baudry B, Savarino SJ, Vial P, Kaper JB, Levine MM (1990) A sensitive and specific DNA probe to identify enteroaggregative Escherichia coli, a recently discovered diarrheal pathogen. J Infect Dis 161: 1249-1251.

13. Nataro JP, Yikang D, Yingkang D, Walker K (1994) AggR, a transcriptional activator of aggregative adherence fimbria I expression in enteroaggregative Escherichia coli. J Bacteriol 176: 4691-4699.

14. Nataro JP, Deng Y, Maneval DR, German AL, Martin WC, et al. (1992) Aggregative adherence fimbriae I of enteroaggregative Escherichia coli mediate adherence to HEp-2 cells and hemagglutination of human erythrocytes. Infect Immun 60: 2297-2304.

15. Yamamoto T, Nakazawa M (1997) Detection and sequences of the enteroaggregative Escherichia coli heat-stable enterotoxin 1 gene in enterotoxigenic E. coli strains isolated from piglets and calves with diarrhea. J Clin Microbiol 35: 223-227.

16. Czeczulin JR, Balepur S, Hicks S, Phillips A, Hall R, et al. (1997) Aggregative adherence fimbria II, a second fimbrial antigen mediating aggregative adherence in enteroaggregative Escherichia coli. Infect Immun 65: 4135-4145.

17. Bernier C, Gounon P, Le Bouguenec C (2002) Identification of an aggregative adhesion fimbria (AAF) type III-encoding operon in enteroaggregative Escherichia coli as a sensitive probe for detecting the AAF-encoding operon family. Infect Immun 70: 4302-4311.

18. Schubert S, Rakin A, Karch H, Carniel E, Heesemann J (1998) Prevalence of the "high-pathogenicity island" of Yersinia species among Escherichia coli strains that are pathogenic to humans. Infect Immun 66: 480-485.

19. Restieri C, Garriss G, Locas MC, Dozois CM (2007) Autotransporter-encoding sequences are phylogenetically distributed among Escherichia coli clinical isolates and reference strains. Appl Environ Microbiol 73: 1553-1562.

20. Boisen N, Ruiz-Perez F, Scheutz F, Krogfelt KA, Nataro JP (2009) Short report: high prevalence of serine protease autotransporter cytotoxins among strains of enteroaggregative Escherichia coli. Am J Trop Med Hyg 80: 294-301.

21. Clermont O, Bonacorsi S, Bingen E (2000) Rapid and simple determination of the Escherichia coli phylogenetic group. Appl Environ Microbiol 66: 4555-4558.
